# Development and internal validation of a novel nomogram for predicting lymph node invasion for prostate cancer patients undergoing extended pelvic lymph node dissection

**DOI:** 10.3389/fonc.2023.1186319

**Published:** 2023-05-08

**Authors:** Zhen Li, Yixin Huang, Diwei Zhao, Xin Luo, Zeshen Wu, Xinyi Zheng, Ye Xie, Yixuan Liu, Jianwei Wu, Yulu Peng, Yonghong Li, Fangjian Zhou

**Affiliations:** ^1^ Department of Urology, Sun Yat-sen University Cancer Center, Guangzhou, China; ^2^ State Key Laboratory of Oncology in Southern China, Collaborative Innovation Center for Cancer Medicine, Guangzhou, China; ^3^ Department of Urology, Huazhong University of Science and Technology Union Shenzhen Hospital (Nanshan Hospital), Shenzhen, China; ^4^ Zhongshan School of Medicine, Sun Yat-sen University, Guangzhou, China; ^5^ School of Clinical Medicine, Tianjin Medical University, Tianjin, China

**Keywords:** Chinese population, lymph node invasion, nomogram, prostate cancer, pelvic lymph node dissection

## Abstract

**Background:**

Few studies have focused on the performance of Briganti 2012, Briganti 2017 and MSKCC nomograms in the Chinese population in assessing the risk of lymph node invasion(LNI) in prostate cancer(PCa) patients and identifying patients suitable for extended pelvic lymph node dissection(ePLND). We aimed to develop and validate a novel nomogram based on Chinese PCa patients treated with radical prostatectomy(RP) and ePLND for predicting LNI.

**Methods:**

We retrospectively retrieved clinical data of 631 patients with localized PCa receiving RP and ePLND at a Chinese single tertiary referral center. All patients had detailed biopsy information from experienced uropathologist. Multivariate logistic-regression analyses were performed to identify independent factors associated with LNI. The discrimination accuracy and net-benefit of models were quantified using the area under curve(AUC) and Decision curve analysis(DCA).The nonparametric bootstrapping were used to internal validation.

**Results:**

A total of 194(30.7%) patients had LNI. The median number of removed lymph nodes was 13(range, 11-18). In univariable analysis, preoperative prostate-specific antigen(PSA), clinical stage, biopsy Gleason grade group, maximum percentage of single core involvement with highest-grade PCa, percentage of positive cores, percentage of positive cores with highest-grade PCa and percentage of cores with clinically significant cancer on systematic biopsy differed significantly. The multivariable model that included preoperative PSA, clinical stage, biopsy Gleason grade group, maximum percentage of single core involvement with highest-grade PCa and percentage of cores with clinically significant cancer on systematic biopsy represented the basis for the novel nomogram. Based on a 12% cutoff, our results showed that 189(30%) patients could have avoided ePLND while only 9(4.8%) had LNI missing ePLND. Our proposed model achieved the highest AUC (proposed model vs Briganti 2012 vs Briganti 2017 vs MSKCC model: 0.83 vs 0.8 vs 0.8 vs 0.8, respectively) and highest net-benefit *via* DCA in the Chinese cohort compared with previous nomograms. In internal validation of proposed nomogram, all variables had a percent inclusion greater than 50%.

**Conclusion:**

We developed and validated a nomogram predicting the risk of LNI based on Chinese PCa patients, which demonstrated superior performance compared with previous nomograms.

## 1 Introduction

Pelvic lymph node dissection (PLND) is routinely performed in patients undergoing prostatectomy for localized prostate cancer (Pca), and the status of pathologically confirmed pelvic lymph node invasion (PLNI) determines the extent of radical prostatectomy (RP) (1). Following the publication of a large sample retrospective clinical report that indicated RP improved the survival of patients with pathologically confirmed PLNI (2), RP has been routinely performed in patients clinically diagnosed with localized prostate cancer regardless of LNI. However, PLND could have been overperformed in the contemporary era because the potential LNI risk in patients with low-risk localized prostate cancer was very low. Even in patients with intermediate and high-risk prostate cancer, most PLND could have been unnecessary due to the low rate of pathologically confirmed LNI, ranging from 8.3% to 12% (3–5).

To avoid unnecessary PLND and identify candidates with LNI for PLND, several nomograms were developed based on patients’ characteristics, such as preoperative prostate-specific antigen (PSA), clinical T stage, and Gleason grade of prostate biopsy (3, 5, 6). Although these nomograms were associated with good performance and underwent external validation (7, 8), they were developed on data from European or American patients. Although the incidence of prostate cancer is much lower in China compared with Western countries, the proportion of patients with metastatic prostate cancer in China is comparatively much higher (4, 9). However, till present, the performance of these published nomograms has not been externally validated in Chinese prostate cancer patients. Further, if their performance is unsatisfactory, a novel nomogram based on clinical characteristics and prostate biopsy pathology of Chinese prostate cancer patients would be required to estimate the risk of LNI.

In this present study, we determined the clinical performance of three existing nomograms, the Briganti 2012, Briganti 2017 and MSKCC (3, 5, 6), in predicting the risk of LNI using a cohort of Chinese prostate cancer patients, then constructed and internally validated a nomogram specifically designed for Chinese prostate cancer patients.

## 2 Patient and methods

### 2.1 Population source and ePLND

With the approval of the institutional review board of our institution, the clinical and pathological data of localized PCa patients who underwent RP and ePLND at the Sun Yat-sen University Cancer Center (SYSUCC, Guangzhou, China) from 2017 to 2021 were retrieved. Patients were excluded if they had received neoadjuvant hormonal therapy. The physician conducted a ultrasound-guided systematic prostate biopsy in accordance with the requirements of section 5.2.7.1.1 of the 2022 EAU guidelines. An experienced uropathologist reviewed all prostate biopsy specimens from the patients.

RP and ePLND were performed by two experienced surgeons (FJ Zhou and YH Li). The ePLND spectrum included the obturator, external iliac, bilateral internal iliac, and common iliac (eight fields) area. The caudal border of ePLND was the deep circumflex vein and the femoral canal, the cranial border was the ureter crossing over the common iliac artery, the lateral border was the genitofemoral nerve, and the medial border was the vesical fat. All fibrofatty tissues along the external iliac vein and within the obturator fossa were removed for harvesting lymph nodes. Lymph nodes along, medial and lateral to the internal iliac vessels were dissected (10). All lymph nodes were then sent for pathological examination in multiple packages according to different surgical fields (3, 11).

### 2.2 Covariates and endpoint

Preoperative PSA, clinical T stage assessed by the attending urologist with extensive experience according to the 2017 American Joint Committee on Cancer staging system (AJCC), was recorded. A digital rectal examination was performed by the attending urologist as the basis for clinical staging. The prostate biopsy data, including the number of cores taken, Gleason score grade group for each positive core, the percentage of positive cores, the percentage of PCa involvement, the percentage of positive cores with highest-grade PCa, the percentage of cores with clinically significant cancer and the percentage of cancer in cores were also recorded. The modified Gleason scoring system based on the 2005 and 2014 International Society of Urological Pathology consensus conferences was used (12, 13). LNI was defined as metastasis in lymph nodes confirmed by pathology.

### 2.3 Statistical analyses

For categorical variables, descriptive statistics were reported on frequencies and proportions. Continuously coded variables were described by the median and interquartile range (IQR). Chi-square and t-test were used to compare proportions and medians of categorical variables and continuously coded variables, respectively. Univariable logistic regression analyses were performed to assessed predictors of LNI. Since Biopsy Gleason grade groups 4 and 5 have a higher degree of malignancy, the Biopsy Gleason grade group was categorized as ≤3 versus ≥ 4. Similarly, clinical stages were categorized as ≥T3 versus ≤T2.

Three well-known nomograms, Briganti 2012, Briganti 2017 and MSKCC nomograms (3, 5, 6), were validated using our cohort. Then, we compared the performance of the 3 nomograms with our developed nomogram in terms of predictive accuracy and net clinical benefit by AUC and DCA, respectively.

Five different models predicting LNI were developed based on the results of univariable logistic regression analyses. Preoperative PSA, clinical T stage, and Biopsy Gleason grade group were included in all 5 models. We quantified the predictive accuracy of multivariable models using the receiver operating characteristic-derived area under the curve (AUC). Given that variables, such as percentage of positive cores and percentage of positive cores with highest-grade PCa, would not improve the predictive accuracy, they were not included in our nomogram. Lastly, preoperative PSA, clinical T stage, biopsy Gleason score group, the maximum percentage of single core involvement with highest-grade PCa, and the percentage of cores with clinically significant cancer were used as the basis of our coefficient-based nomogram. Multivariate logistic model regression coefficients were used to generate our proposed nomogram (SYSUCC model) that could predict the probability of LNI for ePLND in the Chinese cohort. The novel nomogram was conducted with 1000 bootstrap resamples to reduce overfitting bias and perform internal validation. A decision curve analysis (DCA) (12) was performed to determine the net benefit associated with the models. The discrimination and DCA were corrected for overfitting using leave-one-out cross-validation.

All statistical tests were performed using the R statistical package v.3.0.2 (R Project for Statistical Computing, www.r-project.org). All tests were two-sided, with a significance level set at p<0.05.

## 3 Result

### 3.1 Baseline characteristics

A total of 631 PCa patients were eligible for this study, and their descriptive statistics are summarized in **Table 1**. LNI was identified in 194 (30.7%) patients. The median (IQR) number of lymph nodes retrieved was 13 (range, 11-18). Preoperative PSA, clinical T stage, Biopsy Gleason grade group, percentage of positive cores, percentage of positive cores with highest-grade PCa, and percentage of cores with clinically significant cancer on systematic biopsy differed significantly between pN0 and pN1 patients (*p*<0.001; **Table 1**). There were no significant differences in age at surgery and the number of biopsy cores between pN0 and pN1 patients (*p*>0.05; **Table 1**).

**Table 1:** Descriptive statistics of 631 patients with clinically localized prostate cancer (PCa) treated with RP and ePLND from January 2017 to December 2021.

	Overall (n=631)	pN_0_ (n=437)	pN_1_ (n=194)	P value
Age at surgery (yr) Median (IQR)	67.0(62.0,72.0)	67.0(62.0,72.0)	68.0(62.0,71.0)	0.936
Preoperative PSA (ng/ml)Median (IQR)	23.0 (12.5,48.2)	17.6(10.4,31.6)	48.2 (22.9,97.3)	<0.001
DRE(%)	224 (35.5)	123 (28.1)	101 (52.1)	<0.001
Clinical stage (%)				
≤T2	428 (67.8)	335 (76.7)	93 (47.9)	<0.001
≥T3	203 (32.2)	102 (23.3)	101 (52.1)	
Biopsy Gleason grade group (%)				
1	50 (7.9)	46 (10.5)	4 (2.1)	<0.001
2	107 (17.0)	85 (19.5)	22 (11.3)	
3	110 (17.4)	86 (19.7)	24 (12.4)	
4	190 (30.1)	129 (29.5)	61 (31.4)	
5	174 (27.6)	91 (20.8)	83 (42.8)	
No. of cores taken Median (IQR)	11 (10,12)	11 (10,12)	11 (10,12)	0.109
Percentage of positive cores Median (IQR)	50 (20, 83)	42 (18, 67)	82 (50, 100)	<0.001
Percentage of positive cores with highest-grade PCa Median (IQR)	27 (10, 50)	23 (10, 47)	42 (17, 75)	<0.001
Percentage of cores with clinically significant cancer on systematic biopsy Median (IQR)	42 (17, 80)	33 (10, 58)	78 (38, 100)	<0.001
Maximum percentage of single core involvement with highest-grade PCa	60 (40, 80)	50(30, 70)	70 (60, 90)	<0.001
Surgical margin(%)	392 (62.1)	245 (56.1)	147 (75.8)	<0.001
Surgical technique (%)				
ORP	279 (44.2)	153 (35.0)	126 (64.9)	<0.001
RARP	352 (55.8)	284 (65.0)	68 (35.1)	
Gleason grade group at final pathology (%)				
1	22 (3.5)	21 (4.8)	1 (0.5)	<0.001
2	89 (14.1)	79 (18.1)	10 (5.2)	
3	139 (22.0)	111 (25.4)	28 (14.4)	
4	96 (15.2)	66 (15.1)	30 (15.5)	
5	285 (45.2)	160 (36.6)	125 (64.4)	
Pathologic stage (%)				
T2	286 (45.3)	264 (60.4)	22 (11.3)	<0.001
T3a	76 (12.0)	57 (13.0)	19 (9.8)	
T3b	227 (36.0)	106 (24.3)	121 (62.4)	
T4	42 (6.7)	10 (2.3)	32 (16.5)	
Number of removed lymph nodes Median (IQR)	13.0 (11.0,18.0)	13.0(10.0,18.0)	14.0 (12.0, 18.0)	0.017
Number of positive lymph nodes Median (IQR)	0.0 (0.0, 1.0)	NA	2.0(1.0, 4.0)	<0.001

IQR, interquartile range; NA, not applicable; ORP, open radical prostatectomy; PSA, prostate-specific antigen; RARP, robot-assisted radical prostatectomy; PCa, prostate cancer; DRE, the digital rectal examination.

### 3.2 External validation of the Briganti 2012, Briganti 2017 and MSKCC nomograms

External validation of the Briganti 2012 nomogram with a recommended cutoff of 5% revealed that only 1.9% of ePLND needed to be avoided, while 8.3% patients who are not recommend for an ePLND had LNI but missed an ePLND in our cohort. For the Briganti 2017 nomogram with a recommended cutoff of 7%, PLND could be avoided in 7.1% of patients at the cost of missing 6.7% of LNIs in our cohort (**Table 2**). Our results for the MSKCC nomogram, with a recommended cutoff of 2%, showed that no patient could avoid PLND (**Table 2**).

**Table 2:** External validation of Briganti 2012, Briganti 2017 and MSKCC nomogram.

Treatment option	ePLND is not recommended according to the cutoff (below cutoff)	Below cutoff without histologic LNI	Below cutoff with histologic LNI	ePLND is recommended according to the cutoff (above cutoff)	Above cutoff without histologic LNI	Above cutoff with histologic LNI
Briganti 2012 nomogram, 5% cutoff	12(1.9)	11(91.7)	1 (8.3)	619 (98.1)	426 (68.8)	193 (31.2)
Briganti 2017 nomogram, 7% cutoff	45(7.1)	42(93.3)	3(6.7)	586(92.9)	395 (67.4)	191 (32.6)
MSKCC nomogram, 2% cutoff	0(0)	0(0)	0(0)	631(100)	437(69.3)	194(30.7)

LNI, lymph node invasion; ePLND, extended pelvic lymph node dissection.

### 3.3 Establish a novel nomogram predicting LNI

**Figure 1:**
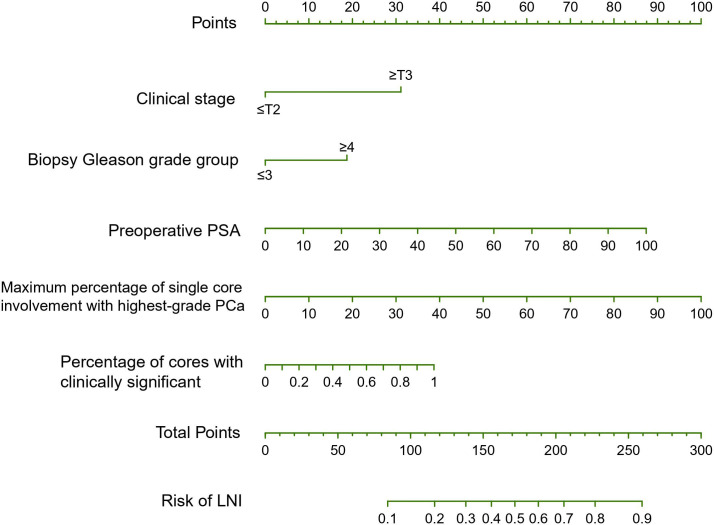
The SYSUCC nomogram predicting the probability of lymph node invasion (LNI) in patients undergoing expend pelvic lymph node dissection (ePLND). PSA, prostate-specific antigen; PCa, prostate cancer.

Based on univariable analyses (**Table 3**), preoperative PSA, clinical T stage, biopsy Gleason grade group, maximum percentage of single core involvement with highest-grade PCa, percentage of positive cores, percentage of positive cores with highest-grade PCa and percentage of cores with clinically significant cancer on systematic biopsy served as predictors of LNI. On multivariable analyses, preoperative PSA, clinical T stage, and biopsy Gleason score group, maximum percentage of single core involvement with highest-grade PCa, percentage of positive cores, and percentage of cores with clinically significant cancer on systematic biopsy presented independent predictors of LNI (**Table 3**). When these covariates were fitted in multivariable models, model 5 (including preoperative PSA, clinical T stage, biopsy Gleason grade group, maximum percentage of single core involvement with highest-grade PCa, and percentage of cores with clinically significant cancer on systematic biopsy) demonstrated the highest AUC on internal validation (83%). Therefore, model 5, termed the SYSUCC model, was chosen as the novel nomogram for predicting LNI in this study. The multivariate influence of each variable on the probability of LNI is visually shown in **Figure 1** as a nomogram. **Supplementary Table 1** illustrates the error and predictive accuracy using the novel nomogram for predicting the risk of LNI when choosing different cutoffs. Using a 12% cutoff, ePLND was estimated to be avoided in 189 of 631 (30%) patients at the cost of missing 4.8%of LNI in patients who harbored LNI. (**Supplementary Table 1**).

**Table 3:** Uni- and multivariable logistic regression analyses assessing predicting the presence of lymph node invasion in 631 patients treated with radical prostatectomy and extended pelvic lymph node dissection.

	Univariable analyses		Model 1 Multivariable analyses		Model 2 Multivariable analyses		Model 3 Multivariable analyses		Model 4 Multivariable analyses		Model 5 Multivariable analyses	
	OR (95% CI)	p value	OR (95% CI)	p value	OR (95% CI)	p value	OR (95% CI)	p value	OR (95% CI)	p value	OR (95% CI)	p value
**Preoperative PSA**	1.03(1.02-1.04)	<0.001	1.03(1.02-1.03)	<0.001	1.02(1.02-1.03)	<0.001	1.02(1.02-1.03)	<0.001	1.02(1.02-1.03)	<0.001	1.02(1.02-1.03)	<0.001
**Clinical stage(≥T3 vs ≤T2)**	3.57(2.49-5.10)	<0.001	2.59(1.74-3.84)	<0.001	2.29(1.52-3.45)	<0.001	2.23(1.48-3.37)	<0.001	2.25(1.49-3.39)	<0.001	2.17(1.43-3.29)	<0.001
**Biopsy Gleason grade group (1-3 vs 4-5)**	2.72(1.88-3.93)	<0.001	2.19(1.45-3.30)	<0.001	1.81(1.18-2.79)	0.007	1.72(1.11-2.65)	0.015	1.81(1.18-2.78)	0.007	1.58(1.01-2.45)	0.044
**Maximum percentage of single core involvement with highest-grade PCa**	1.04(1.03-1.05)	<0.001	–	–	1.03(1.02-1.04)	<0.001	1.03(1.02-1.04)	<0.001	1.03(1.02-1.04)	<0.001	1.03(1.02-1.04)	<0.001
**Percentage of positive cores**	10.45(5.90-18.54)	<0.001	–	–	–	–	2.24(1.14-4.38)	0.019	–	–	–	–
**Percentage of positive cores with highest-grade PCa**	5.45(3.05-9.75)	<0.001	–	–	–	–	–	–	1.57(0.78-3.16)	0.203	–	–
**Percentage of cores with clinically significant** **cancer on systematic biopsy**	12.61(7.23-22.00)	<0.001	–	–	–	–	–	–	–	–	2.98(1.53-5.81)	0.001
**AUC of multivariable models**	–	–	0.79	–	0.82	**-**	0.82	**-**	0.82	**-**	0.83	**-**

PCa, prostate cancer; AUC, area under the curve; PSA, prostate-specific antigen; OR, odds radio.

### 3.4 Comparison of the novel nomogram with currently available models

**Figure 2:**
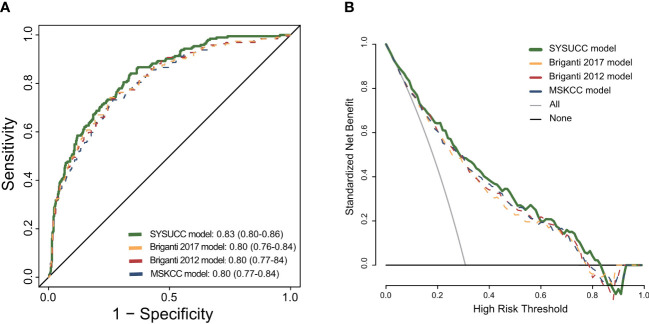
**(A)** Receiver operating characteristic curve for the four prediction models: the SYSUCC model, the Briganti model of 2012, the Briganti model of 2017, the MSKCC model. **(B)** Decision curve analysis (DCA) demonstrating the net benefit associated with use of the SYSUCC model for detection of lymph node invasion (LNI) in comparison to currently three classical models (Briganti 2012, Briganti 2017, and MSKCC model).

The SYSUCC model achieved the highest AUC in our cohort of patients compared with the Briganti 2012, Briganti 2017 and MSKCC models (0.83 vs 0.8 vs 0.8 vs 0.8 for the novel vs Briganti 2012 vs Briganti 2017 vs MSKCC model, respectively) (**Figure 2A**). Compared with the Briganti 2012, Briganti 2017 and MSKCC models, the DCA graphically depicted that the SYSUCC nomogram could improve the clinical risk prediction and had the highest clinical net benefit for LNI threshold probabilities <60% approximately (**Figure 2B**).

At the cutoff of 12%, the SYSUCC nomogram demonstrated the highest ePLND sparing percentage and the lowest percentage of LNIs missing compared with the Briganti 2012, Briganti 2017 and MSKCC nomograms (**Table 4**).

**Table 4:** Clinical implications according to treatment option (SYSUCC nomogram vs Briganti2012 nomogram vs Briganti2017 nomogram vs MSKCC nomogram).

Treatment option	ePLND is not recommended according to the cutoff (below cutoff)	Below cutoff without histologic LNI	Below cutoff with histologic LNI	ePLND is recommended according to the cutoff (above cutoff)	Above cutoff without histologic LNI	Above cutoff with histologic LNI
SYSUCC nomogram, 12% cutoff	189 (30.0)	180 (95.2)	9 (4.8)	442 (70.0)	257 (58.1)	185 (41.9)
Briganti 2012 nomogram, 12% cutoff	158(25.0)	149(94.3)	9 (5.7)	473 (75.0)	288 (60.9)	185 (39.1)
Briganti 2017 nomogram, 12% cutoff	139(22.0)	131(94.2)	8(5.8)	492(78.0)	306(62.2)	186(37.8)
MSKCC nomogram, 12% cutoff	162(25.7)	152(93.8)	10(6.2)	469(74.3)	285(60.8)	184(39.2)

LNI, lymph node invasion; ePLND, extended pelvic lymph node dissection.

### 3.5 Bootstrap validation

All variables had a percent inclusion greater than 50%, which confirmed the stability of these variables in the final model. Besides, the risk ratio with a 95% confidence interval was estimated for each covariate (**Supplementary Table 2**).

## 4 Discussion

PCa patients harbor LNI are at a high risk of poor survival (14, 15). PLND is still the gold standard of nodal staging for detecting LNI in PCa (16). However, expanding the scope of surgery increases the risk of complications (17). Therefore, several clinical guidelines recommend using predictive models developed by clinical data of patients performing ePLND, such as the Briganti 2012, Briganti 2017 and MSKCC nomograms, to assess the risk of LNI in PCa patients and determine who should be considered for ePLND during RP (3, 5, 6). Although these models have high predictive accuracy during internal and external validation in European or American cohorts, their performance in Chinese patients remained undetermined. Given the influence of ethnic differences in PCa, we hypothesized that these models would not be suitable for Chinese PCa patients (18). Thus, we developed and internally validated a novel nomogram predicting LNI specifically based on Chinese PCa patients treated with RP and ePLND and compared it with the three classical models, the Briganti 2012, Briganti 2017 and MSKCC nomograms.

When using our patients to externally validate the three well-established preoperative models (the Briganti 2012, Briganti 2017 and MSKCC nomograms), the AUCs for predicting LNI were substantially lower than those of the cohorts based on which the nomograms were developed. The predictive accuracy of these models was unsatisfactory for application in Chinese settings (**Table 3**). The LNI was 8.3%, 12.0% and 3.7% in the cohorts used for developing the Briganti 2012, Briganti 2017 and MSKCC models, respectively. The LNI in our cohort was 30.7% because the patients had a higher proportion of unfavorable tumor characteristics (4, 9). The performance of the Briganti 2017 (with 12% of LNI), Briganti 2019 (with 12.5% of LNI) and MSKCC (with 3.7% of LNI) nomograms in a recent multicenter cohort (with 24.5% of LNI) was also unsatisfactory (19). These findings suggest that the accuracy of the developed nomograms for predicting LNI differs in cohorts with different PCa grades and stages.

Our SYSUCC model showed better AUC (83%) and net benefit than three well-established Briganti 2012, Briganti 2017, and MSKCC nomograms (**Figure 2**). We attribute this to two new parameters our model updates, compared with the three classical nomograms. First, the results of our univariable analyses show that detailed biopsy characteristics, such as the maximum percentage of single core involvement with highest-grade PCa, which represents a predictor of LNI. Several studies also found out the association between variables that can be considered as a proxy of pathologic tumor volume and the risk of adverse prognosis in PCa patients (20, 21). Meanwhile, after the inclusion of this covariates, the multivariable model 2 shows better improved the accuracy in predicting LNI compared with the base model including PSA, clinical stage, biopsy gleason grade group (**Table 1**, modle 2 vs modle 1). Besides, taking percentage of cores with clinically significant cancer (defined as grade group≥2) on systematic biopsy into consideration can increase the accuracy of our nomogram. The main reason has been the better prognosis associated with Gleason grade group 1 cancer and the more aggressive behavior with Gleason grade group ≥2 cancer (12). One large multi-institutional study demonstrated that a pure Gleason grade group 1 cancer lacks the potential to metastasize to pelvic lymph nodes (22). Therefore, availability of the novel model would improve the predictive accuracy and reduce overestimation of the risk of LNI compared with the three previous models. For example, the calculated risk of LNI in a patient with preoperative PSA 15 ng/ml, T1c, maximum percentage of single core involvement with highest-grade disease 30%, grade group 3 disease in one core, and grade group 1 disease in five out of 12 cores would be higher than 5% according to the Briganti 2012 nomogram、7% according to the Briganti 2017 nomogram and 2% according to the MSKCC nomogram respectively. This patient should be considered for an ePLND according to the EAU-ESTRO-SIOG or NCCN guidelines. However, the results of our model show that this patient would have a risk of LNI lower than 5% and an ePLND could be spared.

Currently, there is no consensus on an optimal nomogram cutoff for determining patients who would benefit most from ePLND. The 2022 NCCN guidelines recommended a 2%cutoff value for the MSKCC nomogram that allows 47.7%of patients to be spared an ePLND at the cost of missing 12.1%patients who harbored LNI. Further, **Table 3** shows that no patients could avoid PLND in our Chinese cohort. The Briganti 2012 and Briganti 2017 also demonstrated comparatively poor performance when using the cutoff the authors recommended, indicating that these cutoffs might not be suitable for Chinese PCa patients. The influence of ethnic differences in the behavior of PCa was reported, and the study population might be a crucial factor in the predictive accuracy of these models (18). Therefore, we had to reevaluate the cutoff value when using the predictive model for a Chinese cohort. We believe the percentage of avoidance should be as high as possible based on a low omission rate. Using a 12% cutoff in our novel model, 189 (30%) patients could avoid ePLND, missing only 9 patients who harbored LNI (4.8%), which is appropriate.

This study had some limitations. First, since this was a single-center study, the findings cannot be generalized to other Chinese institutions. Second, external validation is still needed to confirm the validity of our model for other cohorts. Third, the clinical T stage based on digital rectal examination was used. It has been reported that multiparametric magnetic resonance imaging **(**MRI) based T stage might improve the accuracy of the established models for LNI prediction (8), despite the sensitivity of multiparametric MRI for predicting LNI being about 22.4% (23). Fourth, PSMA-PET-CT was not incorporated into the novel model. The addition of PSMA-PET-CT to the available nomograms (Briganti 2012, Briganti 2017, and MSKCC) was reported to improve the accuracy of the established models for predicting LNI (18), despite the sensitivity of preoperative PSMA-PET/CT for predicting LNI was reported to be <50% (24–26), and PSMA-PET/CT tended to especially miss small lymph node metastases (<5 mm) (27).

Despite these limitations, the proposed SYSUCC model could be regarded as the most appropriate scoring tool for estimating LNI in Chinese PCa patients undergoing RP and judging their need for ePLND. However, further validation is required to confirm these observations.

## 5 Conclusion

The performance of the Briganti 2012, Briganti 2017 and MSKCC nomograms in Chinese patients was unsatisfactory. Thus, the SYSUCC model was established and internally validated for predicting LNI in Chinese PCa patients and promisingly demonstrated that with a cutoff of 12%, ePLND could be potentially avoided in ~30% of patients at the cost of missing 4.8% of LNI. Beseides, based on our novel nomogram, we have set up a website that can easily calculate the risk of LNI. (http://www.sysucc-pca-lni-nomograms.com).

## Data availability statement

The datasets presented in this article are not readily available because of the data sharing policy and procedures of the institution and groups who conducted the original study. Requests to access the datasets should be directed to the corresponding authors.

## Ethics statement

The studies involving human participants were reviewed and approved by the institutional review board (IRB) and the ethical committee of Sun Yat-sen University Cancer Center. Written informed consent for participation was not required for this study in accordance with the national legislation and the institutional requirements.

## Author contributions

Writing original draft: ZL YXH; data curation: DWZ, XL; study design and methodology: YLP, YHL and FJZ; statistical analysis and data visualization: XL, YXH, and DWZ; project conceptualization: DWZ, ZSW, XYZ, YX, JWW and YXL; project execution, supervision, and administration: YH L and FJZ.
