# Supplementary material for: Development and internal validation of a novel nomogram for predicting lymph node invasion for prostate cancer patients undergoing extended pelvic lymph node dissection
Source: Front Oncol. 2023 May 8;13:1186319. doi: 10.3389/fonc.2023.1186319 (PMC10202171; doi:10.3389/fonc.2023.1186319)
Supplement: Supplementary file 1 [file Table_1.docx]

**Supplement Table 1 Systematic analyses of the SYSUCC nomogram-derived cutoffs used to discriminate between patients with or without histologically confirmed lymph node invasion.**

| Calculated  probability of LNI, cutoff (%) | ePLND is not recommended  according to the cutoff  (below cutoff) | Below cutoff without histologic  LNI | Below  cutoff  with  histologic  LNI | ePLND is recommended according to the cutoff  (above cutoff) | Above cutoff without histologic LNI | Above  cutoff  with  histologic  LNI |
| --- | --- | --- | --- | --- | --- | --- |
| 5 | 66 (10.5) | 65 (98.5) | 1 (1.5) | 565 (89.5) | 372 (65.8) | 193 (34.2) |
| 10 | 157 (24.9) | 149 (94.9) | 8 (5.1) | 474 (75.1) | 288 (60.8) | 186 (39.2) |
| 11 | 172 (27.3) | 164 (95.3) | 8 (4.7) | 459 (72.7) | 273 (59.5) | 186 (40.5) |
| 12 | 189 (30.0) | 180 (95.2) | 9 (4.8) | 442 (70.0) | 257 (58.1) | 185 (41.9) |
| 13 | 203 (32.2) | 192 (94.6) | 11 (5.4) | 428 (67.8) | 245 (57.2) | 183 (42.8) |
| 14 | 223 (35.3) | 207 (92.8) | 16 (7.2) | 408 (64.7) | 230 (56.4) | 178 (43.6) |
| 15 | 237 (37.6) | 217 (91.6) | 20 (8.4) | 394 (62.4) | 220 (55.8) | 174 (44.2) |
| 16 | 243 (38.5) | 221 (90.9) | 22 (9.1) | 388 (61.5) | 216 (55.7) | 172 (44.3) |
| 17 | 258 (40.9) | 233 (90.3) | 25 (9.7) | 373 (59.1) | 204 (54.7) | 169 (45.3) |
| 18 | 270 (42.8) | 244 (90.4) | 26 (9.6) | 361 (57.2) | 193 (53.5) | 168 (46.5) |
| 19 | 275 (43.6) | 249 (90.5) | 26 (9.5) | 356 (56.4) | 188 (52.8) | 168 (47.2) |
| 20 | 285 (45.2) | 258 (90.5) | 27 (9.5) | 346 (54.8) | 179 (51.7) | 167 (48.3) |
| 25 | 330 (52.3) | 293 (88.8) | 37 (11.2) | 301 (47.7) | 144 (47.8) | 157 (52.2) |
| 30 | 371 (58.8) | 325 (87.6) | 46 (12.4) | 260 (41.2) | 112 (43.1) | 148 (56.9) |

LNI = lymph node invasion; ePLND = extended pelvic lymph node dissection

**Supplement Table 2 Percent Inclusion of Each Variable in Variable Selection Step and Results of Parameter Estimation Step of Bootstrap Validation**

|  | **Risk Ratio** | **95% CI** | **Percent** |
| --- | --- | --- | --- |
| **Clinical stage** | 2.244 | 2.222-2.265 | 0.975 |
| **Biopsy Gleason grade group** | 1.602 | 1.576-1.628 | 0.521 |
| **Preoperative PSA** | 1.021 | 1.021-1.022 | 1 |
| **Maximum percentage of single core involvement with highest-grade PCa** | 1.026 | 1.025-1.026 | 1 |
| **Percentage of cores with clinically significant**  **cancer on systematic biopsy** | 2.731 | 2.681-2.782 | 0.745 |

PCa = prostate cancer; PSA = prostate-specific antigen
